# Building a cognitive screening and assessment pathway for primary care: The experience of one health care system

**DOI:** 10.1002/alz70860_100447

**Published:** 2025-12-23

**Authors:** Kyra O’Brien, Wheeler Maxwell, Robert Burke, Joseph Teel, Jason Karlawish

**Affiliations:** ^1^ University of Pennsylvania Perelman School of Medicine, Philadelphia, PA, USA

## Abstract

**Background:**

Penn Medicine's Penn Memory Center (PMC) provides top‐tier diagnosis and care for patients with Alzheimer's disease and related dementias. Unfortunately, a long wait for new patient evaluations limits access to the PMC. The result is delayed diagnoses and care. One way to address this is to diagnose patients earlier in the primary care setting, where patients typically first present with cognitive complaints. There are numerous barriers to detecting cognitive impairment in primary care, including limited time and competing priorities. Building on our research identifying and examining these barriers, we will select and implement interventions to improve detection of cognitive impairment in primary care and facilitate timely and appropriate referrals for care. This process will be supported by the Davos Alzheimer's Collaborative (DAC) U.S. Early Detection Fellowship Program.

**Method:**

Clinician and operations stakeholders from neurology, geriatrics, and primary care selected strategies to facilitate detection of cognitive impairment in primary care via a modified Delphi consensus process. We will pilot selected strategies with support from the DAC Early Detection Blueprint, implementation coaches, and collaboration with other Fellowship Program sites. Key outcomes include measures of feasibility and acceptability of the intervention via interviews and surveys of primary care providers and other clinical staff engaged in intervention delivery and changes in use of standardized cognitive assessments and rates of detection of cognitive impairment. An implementation team will iteratively assess outcomes and refine the strategies to facilitate sustainability and dissemination.

**Result:**

The multidisciplinary stakeholder panel selected six strategies for implementation by a. A cognitive assessment algorithm for primary care was chosen as the first intervention to pilot, including a cognitive screen (the Linus Health Digital Clock and Recall) to be performed during the Medicare Annual Wellness Visit. We are in the planning phase for piloting the cognitive screen. The cognitive assessment pathway was designed with input from clinical and operations stakeholders and will be implemented as an electronic health record‐embedded clinical pathway via AgileMD.

**Conclusion:**

The Early Detection Blueprint and other resources of the Fellowship Program support the implementation of a cognitive impairment detection program for primary care, designed for dissemination across Penn Medicine and similar health systems.

